# High fat diet causes distinct aberrations in the testicular proteome

**DOI:** 10.1038/s41366-020-0595-6

**Published:** 2020-07-16

**Authors:** S. Jarvis, L. A. Gethings, L. Samanta, S. M. A. Pedroni, D. J. Withers, N. Gray, R. S. Plumb, R. M. L. Winston, C. Williamson, C. L. Bevan

**Affiliations:** 1 Department of Surgery and Cancer, Imperial College, Hammersmith Hospital London, London, UK; 2 Waters Corporation, Wilmslow, UK; 3 American Center for Reproductive Medicine, Cleveland Clinic, 10681 Carnegie Avenue, Desk X11, Cleveland, OH 44195 USA; 4 Redox Biology Laboratory, Department of Zoology, School of Life Sciences, Ravenshaw University, Cuttack, Odisha 753003 India; 5 MRC London Institute of Medical Sciences, London, United Kingdom; 6 Institute of Clinical Sciences, Faculty of Medicine, Imperial College London, London, W12 0NN UK; 7 Division of Computational and Systems Medicine, Sir Alexander Fleming Building South Kensington, London, SW7 2AZ UK; 8 Waters Corporation, Maple Street, Milford, MA USA; 9 Department of Women and Children’s Health, Kings College London, Guys Campus, London, UK

**Keywords:** Proteins, Obesity

## Abstract

Diet has important effects on normal physiology and the potential deleterious effects of high fat diets and obesity on male reproductive health are being increasingly described. We conducted a histological review of the effects of chronic high fat (HF) diet (using a mouse model fed a 45% fat diet for 21 weeks) with a discovery proteomic study to assess for changes in the abundance of proteins in the testis. Mice on a HF diet became obese and developed glucose intolerance. Using mass spectrometry, we identify 102 proteins affected in the testis of obese mice. These included structural proteins important for the blood testis barrier (filamin A, FLNA), proteins involved in oxidative stress responses (spermatogenesis associated 20, SPATA-20) and lipid homoeostasis (sterol regulatory element-binding protein 2, SREBP2 and apolipoprotein A1, APOA1). In addition, an important regulator protein paraspeckle component 1, PSPC-1, which interacts with the androgen receptor was significantly downregulated. Proteomic data was validated using both Western blotting and immunostaining which confirmed and localised protein expression in both mouse and human testis using biopsy specimens. This study focused mainly on the abnormalities that occurred at the protein level and as a result, we have identified several candidate proteins and conducted pathway analysis around the effects of HF diet on the testis providing novel insights not previously described. Some of the identified targets could be targeted therapeutically and future work is directed in this area.

## Introduction

Diet can have a significant impact on normal physiology, with high calorific foods and sedentary lifestyles contributing to the development of obesity, an epidemic in adults, as well as children [1]. Obesity comes with a plethora of adverse health consequences, including cardiovascular diseases, dyslipidaemias, non-alcoholic fatty liver disease and higher incidence of type 2 diabetes and various cancers including female cancers [2–5]. It is also increasingly recognised that being overweight or obese may have a deleterious effects on female fertility [6, 7]. Men with higher body mass index (BMI) are more likely to have unfavourable semen parameters with a higher incidence of azoospermia [8–10]. Diets high in saturated fat are negatively correlated with sperm concentration and this has been described to occur in a dose dependent manner [11].

The association between obesity and impaired male reproductive function is multifactorial, involving alterations at the level of the hypothalamic-pituitary-gonadal (HPG) axis, as well as direct testicular effects on spermatogenesis and somatic cell function [12]. Rodent studies which use high fat (HF) diet to induce obesity reveal reductions in sperm volume and motility with a higher percentage of morphologically abnormal sperm [13]. Obesity may also increase testicular vulnerability to environmental insults and subfertility from pathophysiological states (e.g., cholestasis) [14–16]. HF diets affects androgen receptor (AR) expression essential for male fertility [17] also causing testicular inflammation, increased oxidative stress causing sperm DNA damage [18, 19]. Effects continue after conception affecting embryo quality and implantation rates [20]. Furthermore, HF diet may also affect the sperm epigenome with trans-generational effects to the offspring of obese males [18, 21].

However, key aberrations in gene targets or pathways in the testis from HF diets are still relatively unknown with modest changes in RNA transcripts in the mouse testis making it challenging to understand pathways by which HF diets exerts such deleterious consequences [18, 22, 23]. Gene expression studies are unlikely to be the most relevant investigation to comprehensively understand the effects of HF on the testis, particularly as male germ cells become transcriptionally silent in late spermatogenesis. At the protein level, one recent study used proteomic analysis in conjunction with long non-coding RNA arrays to study testes from rats fed a HF diet; cytoskeleton changes and oxidative stress were found to be important [24].

Here, we used a discovery approach to study the effects of diet-induced obesity on the testis, generating a valuable proteomic dataset of potential ‘hits’ providing insight into how obesity may affect specific biological pathways and protein networks in the testis.

## Methods

### Animal experiments

In vivo studies were performed in accordance with the United Kingdom Animals (Scientific Procedures) Act (1986) and Imperial College’s Animal Welfare and Ethical Review Body. 3Rs approach was followed in these studies and we obtained testicular tissues from wild type C57BL/6 mice, used for other HF studies [25]. We undertook a comparison of the tissues between male mice randomised to either a diet consisting of 45% fat (HF) diet (Research Diets, New Brunswick NJ Cat# D1245) or standard mouse chow (NC) (4.25% fat, RM3; Special Diet Services) between 11 and 32 weeks of age. They were maintained on a 12-h light/dark cycle with free access to water and housed in specific-pathogen free barrier facilities. Testes were isolated, flash frozen in liquid nitrogen and processed for relevant studies.

### Human testicular biopsies sample collection

Human testicular biopsies were obtained from consented patients undergoing microsurgical extraction of sperm (mTESE) procedures for sperm retrieval (London REC reference 05/q0406/159). Samples were processed for histological assessment and immunofluorescence staining.

### Assessment of weight and glycaemic status of mice

Mice were weighed from 4 weeks of age. At 8 and 27 weeks of age, an intra-peritoneal glucose tolerance test (ipGTT) was performed. After an overnight fast, mice were given an intra-peritoneal injection of glucose (2 mg/kg-20% v/w) and blood samples collected from the tail vein immediately prior to the injection (time 0) and 15, 30, 60 and 120-minutes after glucose administration.

### Histological analysis and immunostaining

Testicular sections from 4 mice per group were analysed for stereological estimates and immunohistochemistry. Testes were fixed in Bouin’s fluid (Sigma Aldrich) or 10% Neutral buffered formalin (Fisher Scientific) at 4 °C overnight and treated through graded ethanol and Histoclear (National Diagnostics Ltd) ready for paraffin embedding. Serial sections (5–6 µm) were stained with a standard haematoxylin and eosin (H&E) and counting methods undertaken as previously described to quantitate spermatogenesis [26–29]. In addition, Immunofluorescence and Terminal deoxynucleotidyl transferase staining (TUNEL) staining (Roche, UK) performed with Zeiss 510 confocal microscopy for visualisation (see supplementary methods).

#### Western blotting

Whole mouse testes were disrupted in 1 ml of Tissue PE lysis buffer (G Biosciences) and protease cocktail inhibitor (Sigma) using Qiagen TissueLyser. After protein quantification with appropriate standards (Biorad**™** DC Protein Assay), 10 μg protein extract was separated using SDS-PAGE. Gels were electroblotted onto Immobilon-P membranes (Millipore) using semi-dry transfer (Bio-Rad Trans-Blot SD^®^). Non-specific binding was blocked using 5% w/v non-fat dried milk diluted in Phosphate Buffered Saline plus 0.05% Tween-20 (PBS-T). Primary antibodies were mouse monoclonal against β-actin at [1:5000], filamin A [1:1000], paraspeckle protein-1 [1:1000] (Abcam, Cambridge, MA, USA) and SPATA-20 [1:1000] (Atlas Antibodies). All membrane washes were undertaken with PBS-T. Peroxidase-labelled rabbit anti-mouse and goat anti-rabbit secondary antibodies (Dako, Ely, UK) were used at 1:2000. Membranes were incubated with Luminata Forte Western HRP substrate for 10 min (Millipore) followed by detection of chemiluminescence and photo acquisition (Fusion Solo, Vilber).

#### Preparation of samples for proteomic studies

##### Protein extraction protocol

Frozen testicular tissue (40 mg) was thawed and placed in pre-chilled bead-beater tubes containing 100 µL 1 mm zirconium beads. Protein extraction was conducted as previously reported [30]. A Precellys bead beater was used to homogenise the samples at 6,500 Hz (2 × 40 s cycles), in 100 mM Tris-HCL (pH 7.6), 100 mM DDT and 4% SDS lysis buffer and samples incubated at 95 °C for 3 min, sonicated for 5 min before centrifugation at 15,000 × *g* for 10 min at 20 °C. Supernatant was removed and protein concentration determined prior to analysis using BCA-assay (Thermo Scientific, Loughborough, UK) following the manufacturer’s protocol.

##### Protein digestion

Testicular homogenates were diluted to ~1 mg/ml in 0.1% (w/v) RapiGest (Waters Corporation, Milford, USA) in 50 mM ammonium bicarbonate (Sigma Aldrich, St. Louis, MO) and heated at 80 °C for 45 min. Samples were reduced with 5 mM dithiothreitol (Sigma Aldrich) at 60 **°**C for 30 min and alkylated with 15 mM iodoacetamide (Sigma Aldrich) at room temperature in the dark for 30 min. Proteolytic digestion was undertaken by adding sequencing grade TMPK-treated trypsin gold, mass spectrometry grade (Promega, Madison, MI) at a 1:25 (w/w) ratio and incubation overnight at 37 °C. Trifluoroacetic acid (Sigma Aldrich) was added to a final concentration of 0.5% (v/v) to hydrolyse the RapiGest and the solutions incubated at 37 °C for 20 min before vortexing and centrifugation steps (10 min at 10,000 rpm). Supernatant was diluted with water containing 0.1% (v/v) formic acid.

##### LC-MS configuration

1D nanoscale LC separation of tryptic peptides was performed with the ACQUITY M Class system (Waters Corporation), configured with a Symmetry C18 5 µm, 2 cm × 180 µm pre-column and an HSS T3 C18 1.8 µm, 25 cm × 75 µm analytical RP column (Waters Corporation). MS analysis of tryptic peptides was performed using a Synapt G2-S*i* mass spectrometer (Waters Corporation, Wilmslow, UK) (further information given in supplementary methods). Samples were transferred with aqueous 0.1% (v/v) formic acid to the precolumn at a flow rate of 5 µl/min for 5 min. Mobile phase A consisted of water containing 0.1% (v/v) formic acid, whilst mobile phase B consisted of acetonitrile containing 0.1% (v/v) formic acid. After desalting and preconcentration, peptides were eluted from the pre-column to the analytical column with separation using a gradient of 3-40% mobile phase B for 90 min (flow rate of 300 nl/min), followed by a 2 min column rinse with 85% of mobile phase B. Re-equilibration of columns used initial conditions for a 20 min period. Analytical column temperature was maintained at 35 **°**C and lock mass compound, [Glu^1^]-Fibrinopeptide B (Sigma-Aldrich, St Louis, MO)(200 fmol/µl), was delivered by the auxiliary pump of the LC system at 500 nl/min to the reference sprayer of the NanoLockSpray source of the mass spectrometer. Accurate mass LC-MS data were collected in mobility assisted data independent (LC-UDMS^E^) mode of acquisition [31]. MS analysis of tryptic peptides was performed using a Synapt G2-S*i* mass spectrometer (Waters Corporation, Wilmslow, UK). All analyses were performed in positive mode electrospray ionisation (ESI) and time of flight analyser (ToF) externally calibrated with a NaCsI mixture from *m/z* 50 to 1990. The reference sprayer was sampled with a frequency of 60 s. Accurate mass LC-MS data were collected in mobility assisted data independent (LC-UDMS^E^) mode of acquisition [31].

Progenesis QI for proteomics (Nonlinear Dynamics, Newcastle upon Tyne, UK) was used to peak pick, align, normalise and provide relative quantification from the acquired proteomic data. Protein identifications were obtained by searching the reviewed entries of *Mus musculus* UniProt database (16,738 entries, release 2016_02). Median abundance normalisation was conducted using a two-group experimental design each consisting of data from 3 mice per group (with 3 technical replicates per samples). Relative protein abundance levels and associated reliability of the measured differences, were considered significant with a minimum fold change of two between both biological groups and an ANOVA *p*-value ≤ 0.05. Protein identifications were further curated on the basis of replication and accepted if consistently found in ≥2 biological and technical replicates.

##### Gene ontology (GO) search and protein:protein interactions

GO terms were generated for biological and molecular process by uploading the top differentially expressed proteins between the HF and normal chow (NC) fed mice using PantherDB (Protein ANalysis THrough Evolutionary Relationships, version 11) [32]. Protein–protein interactions between differentially expressed proteins in HF conditions were determined using STRING (version 10.5) [33].

##### Ingenuity pathway analysis (IPA)

Ingenuity Pathway Analysis (IPA®, QIAGEN Redwood City) was used for pathway analysis and top canonical pathways are presented which were affected by HF diet using Fisher’s exact test.

## Results

### High fat diet resulted in increased body weight and insulin resistance

**Fig. 1:**
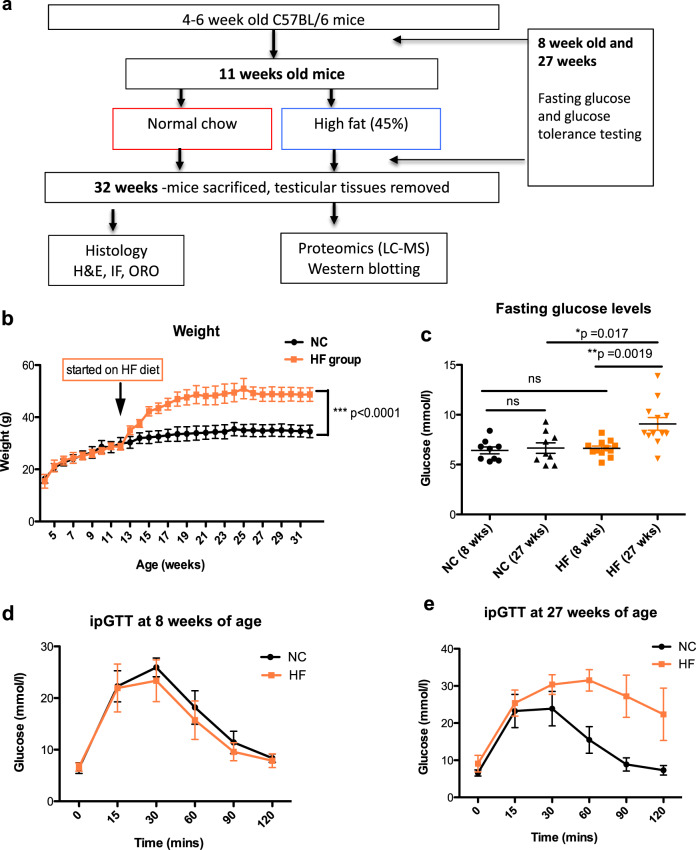
Effects of the HF diet on body weight and glycaemic control. **a** Experimental set up and use of tissues. **b** Body weight. Mice were weighed weekly prior to and after receiving the HF (*n* = 9) or NC diets (*n* = 7). **c** Effect of HF diet on fasting glucose when mice were tested at 8 weeks of age when all were on the same diet and then repeated at 27 weeks of age (16 weeks after commencing HF diet) or ongoing NC diet. **d** Effect of HF diet on glucose and (**e**) glucose tolerance. Statistical analysis performed using 2-way ANOVA ***p < 0.001 comparing HF to control. Abbreviations Oil red O (ORO), Haematoxylin & Eosin (H&E), Immunofluorescence (IF), intraperitoneal glucose tolerance test (ipGTT).

The effects of diet on long-term body weight regulation in male mice fed either a normal chow (NC) or high-fat (HF) diet was studied (Fig. 1a) and mice weight and glucose tolerance represents further analysis of previously published data [25]. At 11 weeks of age, average body weight for all mice was 28 ± 2.31 g (mean ± SD). High fat (HF) diet was introduced to the experimental group from 11-weeks of age (*n* = 13 mice) whilst the control group continued with standard normal chow (NC) diet (*n* = 9). After 2 weeks of the HF diet mice gained +5.45 ± 2.34 g, compared to +1.83 ± 0.64 g in the control group. By the end of the study when mice were 32 weeks of age, those on the HF diet were significantly heavier (by ~12 g) than those on the NC diet, weighing 46.77 ± 6.14 g compared to 34.47 ± 2.85 g in the control group (*p* < 0.0001) (Fig. 1b).

Fasting glucose levels were measured before and after mice were exposed to either HF or NC conditions (Fig. 1c). At baseline testing at 8 weeks of age, there were no differences in the fasting glucose levels between mice which remained on NC diet and those that were subsequently allocated to the HF diet (Fasting glucose levels of 6.42 ± 1.04 mmol/l and 6.63 ± 0.79 mmol/l, respectively). At 27 weeks, when mice in the HF group had received 16 weeks of HF diet, this group had significantly higher (**p = 0.011) fasting glucose levels (9.08 ± 2.22 mmol/l) compared to mice on the NC diet (6.65 ± 1.58 mmol/l). Intraperitoneal glucose tolerance tests (ipGTT) at the same timepoints showed that there was significant deterioration in glucose tolerance in mice on the HF compared to NC diet (*p* < 0.0001) (Fig. 1d, e), which suggested establishment of the insulin resistance associated with the obesity phenotype.

### HF diet alters Sertoli cell and mature germ cells in the testis

**Fig. 2:**
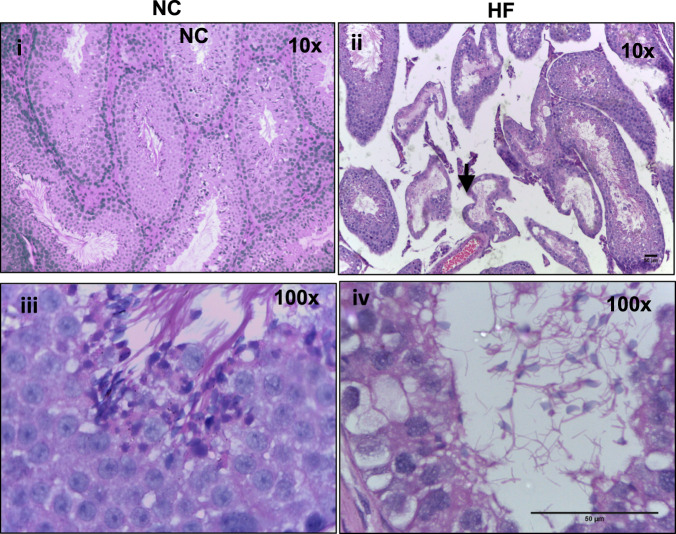
Histological alterations in response to HF diet in the mouse testes. Representative histological images of H&E stained testicular tissues from mice fed either a HF diet (I,iii) as compared to control mice fed a NC diet (ii, iv) at the end of study (aged 32 weeks). Narrowing of seminiferous tubules is highlighted (black arrow).

At 32 weeks of age, testicular tissues were collected for histological assessment. The HF experimental group exhibited gross narrowing of the seminiferous tubules (*p < 0.05) (Fig. 2). Reduced Sertoli cell numbers, meiotic index and numbers of post-meiotic round spermatids were observed (Supplementary Table 1). Apoptosis was not increased in the tissues from HF mice (Supplementary Fig. 1).

### Proteomic data

**Fig. 3:**
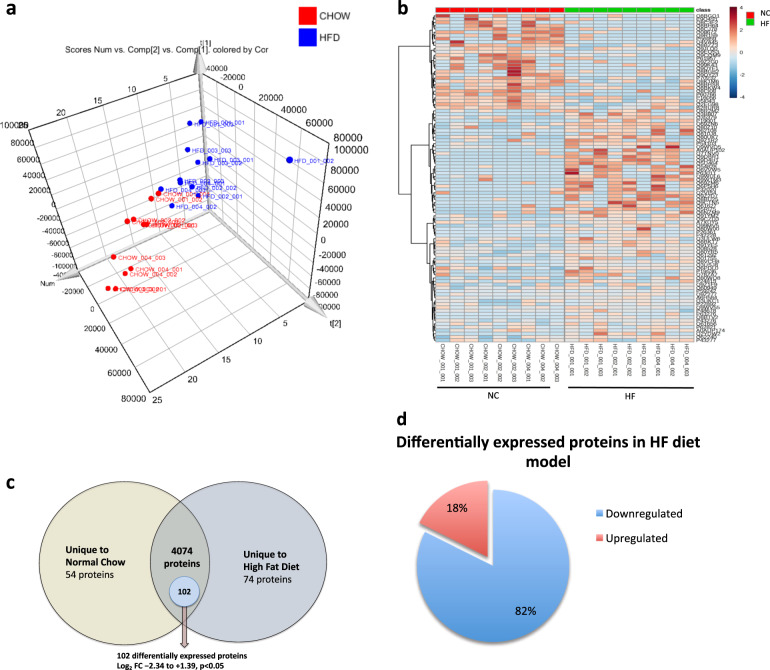
Summary of proteomic data after multivariate analysis with depiction of differentially expressed proteins in the testes between HF and NC conditions. **a** Multivariate analysis of proteomic data demonstrating 3D scores plot from unsupervised principal components analysis (PCA) showing separation between the testicular tissues from mice fed a HF diet compared to NC. **b** Hierarchal cluster heatmap based on protein expression between HFD vs. Chow mice and the contribution to the separation of phenotypes by unique protein IDs to either HF or NC diets. Data is presented as technical triplicates for each mouse sample analysed (*n* = 3). Each lane represents a technical triplicate (Created in Metaboanalyst v3.0). **c** Venn diagram representation of proteins in the testis between the HF and NC conditions. 1012 proteins were found uniquely in NC mouse testes whilst 920 were uniquely in HF mice. 3028 proteins were expressed in both conditions of which 102 proteins were significantly altered in HF testes. The Log2 FC fold change for these 102 proteins ranged between −2.34 and 1.39 (*p* < 0.05). **d** Direction of the differentially expressed statistically significant proteins in HF condition (*n* = 102 proteins).

We investigated global differences in protein expression between the two experimental conditions. Firstly, unsupervised principal components analysis (PCA) and hierarchal heatmap clustering showed separation between protein signatures of testes acquired from mice on HF versus NC diets (Fig. 3a, b). In total, 4960 proteins were identified based on a minimum of 1 peptide per protein (identifications have greater specificity using ion mobility as part of the acquisition) and a 1% FDR. From the identified proteins, 54 were detected only in the testes from mice fed NC whilst 74 were unique to the testes from mice on the HF diet (Fig. 3c). Quantitative comparison of the remaining proteins expressed in both conditions (*n* = 4074 proteins) revealed that 2.2% of these proteins (*n* = 102) were statistically deregulated by HF diet (*p* < 0.05) with a log2 fold change between −2.34 and +1.39 (Supplementary Table 2). Protein quantification was performed using only unique peptides per protein. Of the differentially expressed proteins (*n* = 102), 82% were downregulated, whilst 18% were upregulated (Fig. 3d).

All proteins were functionally annotated for GO terms using PANTHER. The distribution of these proteins in relation to GO biological processes is shown (Supplementary Table 3) and revealed clusters of proteins related to meiosis, mitosis and chromosomal segregation. In addition, roles related to catabolism, protein transport and cellular biogenesis were identified.

**Fig. 4:**
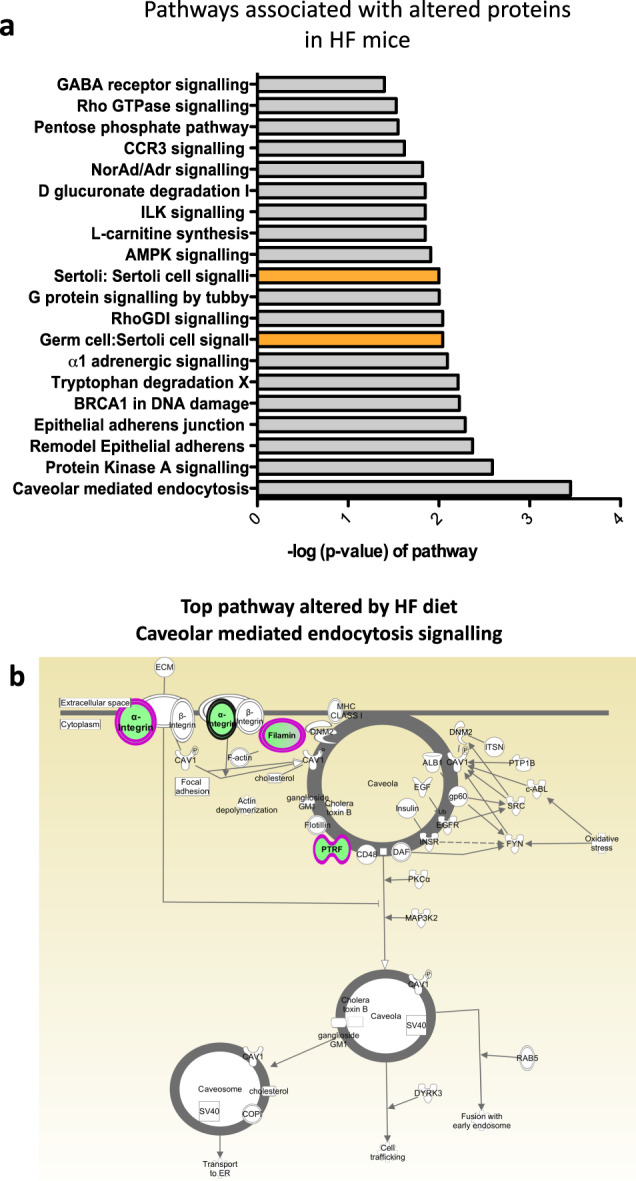
Top IPA pathways deregulated in the testes in HF diet versus NC conditions. **a** Top Ingenuity pathways associated with altered proteins in the testes from mice fed a HF diet as compared to NC mice. Bars indicate the likelihood [−log (*P*-value)] that the specific pathway is affected by dietary fat. Protein IDs generated after LC-MS/MS analysis of the global testicular proteome in HF and NC fed mice were put through IPA to generate a total number of curated proteins relevant to that pathway identified in the whole testis. The *p* value represents the pathway significance to the tissue. Testis specific pathways related to Germ cell-Sertoli cell junction signalling; Germ cell:Germ cell signalling are highlighted in orange. **b** Top pathway downregulated in testis from HF conditions: caveolar mediated endocytosis signalling, green depicts downregulated proteins in the pathway including FILAMIN A, FILAMIN B, ITGA11 (alpha integrin subunit 11), PTRF/CAV1 (caveolae associated protein).

All protein IDs identified from the mass spectrometry data which were significantly and differentially expressed in the HF diet tissues were converted into protein names using UniprotKB (version 2018_07). In order to understand the biological pathways over represented by these proteins, Ingenuity pathway analysis was undertaken (using the 102 proteins differentially expressed from the HF diet model) (Fig. 4a). The top disrupted pathway in mice fed a HF diet was caveolar mediated endocytosis signalling (Fig. 4b) which had the highest pathway score. Other pathways included epithelial adherens signalling and remodelling, protein kinase A and Rho GDI signalling. Testis-specific pathways disrupted in mice fed a HF diet included Germ cell:Sertoli cell junction signalling and Sertoli:Sertoli cell signalling. Also, sperm motility proteins were disrupted but this fell outside of the top 20 disrupted pathways.

A protein–protein interaction network was constructed using the STRING database. Network analysis confirmed that a large proportion of the 102 differentially expressed proteins altered by HF diet were functionally connected and that the network of proteins had significantly more interactions than would be expected by chance (*P* value = 0.0036) (Supplementary Fig. 2).

### Validation of proteomic data

To validate the proteomic data, Western blotting was undertaken using testicular protein lysates from the same mice used for proteomic experiments [34]. Proteins chosen for validation were selected on the basis of (i) magnitude of fold change and (ii) likely biological relevance to fertility. Five proteins altered by HF diet from the proteomic dataset were selected (paraspeckle component 1 [PSPC-1], sterol regulatory element-binding protein 2 [SREBP2], filamin A [FLNA], apolipoprotein A1 [APOA1] spermatogenesis associated 20 [SPATA-20]) (Supplementary Table 4).

**Fig. 5:**
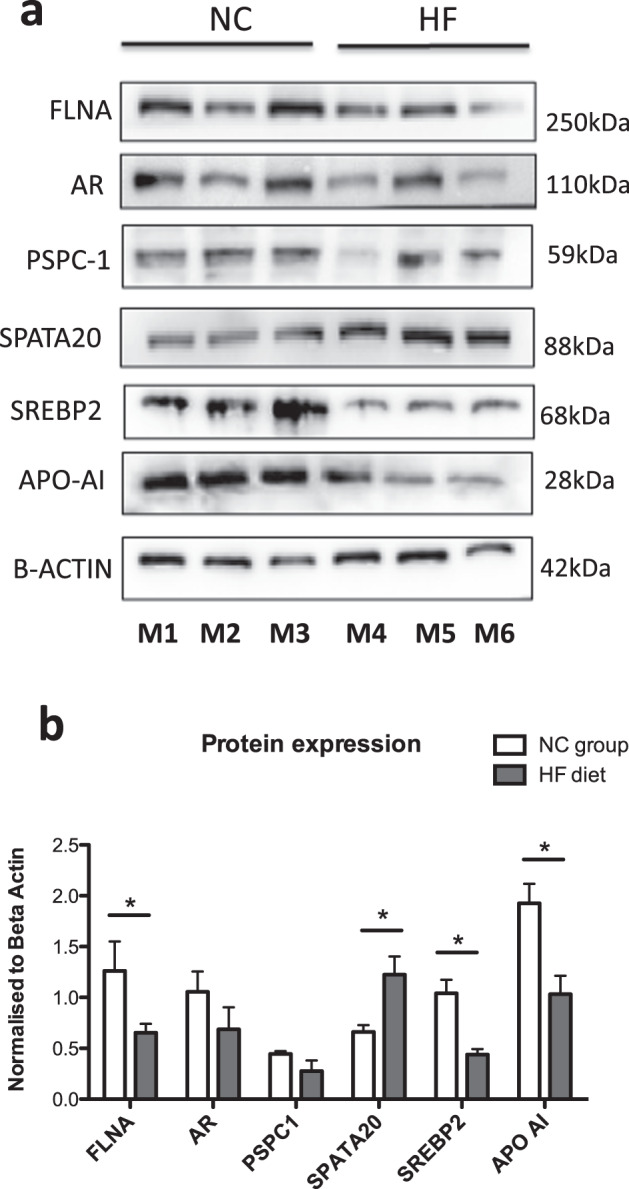
Western blot validation studies of 6 key protein targets differentially expressed in the testes from mice on HF diet versus NC conditions. **a** HF diet decreases the protein expression of FLNA, AR and PSPC-1, SREBP2, APOA-I with an increased in SPATA20 expression. **b** Western blots of all proteins extracted from testicular tissues from 3 representative mice and (**c**) semi-quantitative protein expression using band densitometry analysis using image J. Values are average +/−SEM (*n* = 3 per group). *p < 0.05, **p < 0.01, ***p < 0.001 and compared using by two tailed unpaired *t* test.

Western blots analysis confirmed downregulation of SREBP2 and APOA, FLNA and PSPC-1 (Fig. 5). Previous studies have shown that androgen receptor (AR) is downregulated in testis in the context of HF conditions [17]. We also confirmed reduced AR in the HF conditions by Western blotting, although this was not statistically significant by band densitometry.

Lastly, SPATA-20, thioredoxin-like protein was investigated as an important protein upregulated in testicular oxidative stress (associated with HF diet and obesity) with increase in SPATA-20 noted thus validating LC-MS proteomic findings (Fig. 5).

**Fig. 6:**
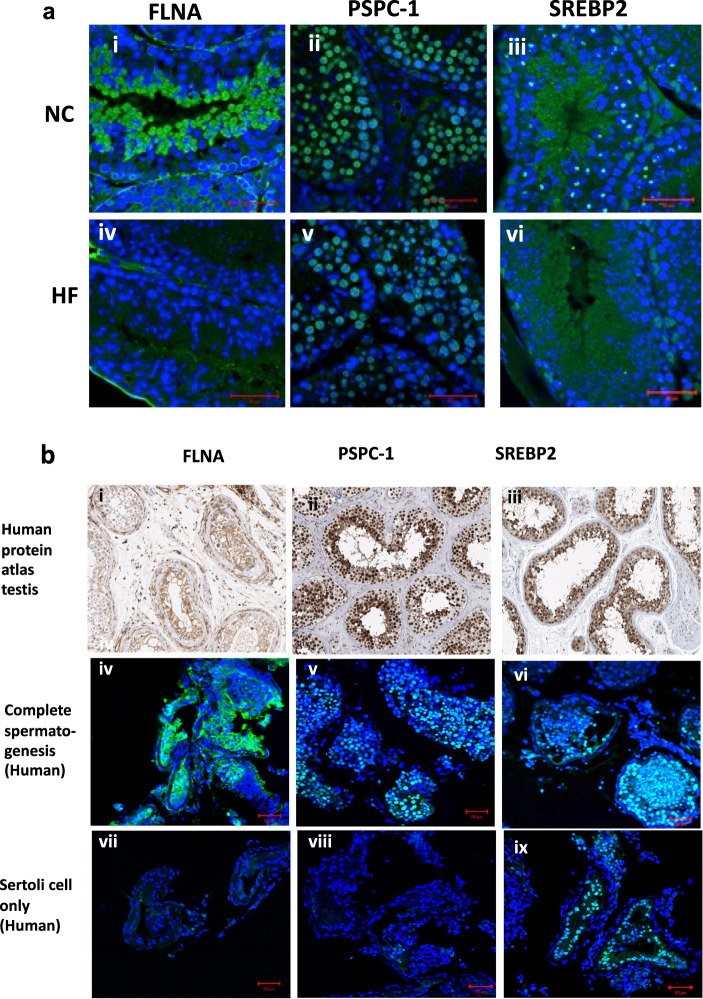
Immunofluorescence imaging comparing altered protein targets (FLNA, PSPC- 1, SREBP2) between representative testes from mice fed NC and HF diet and expression in human testis. Representative immunofluorescence images comparing altered protein targets (FLNA, PSPC-1, SREBP2) between representative testes from (**a**) mice fed NC and HF Mouse testis. All images at presented at ×63, scale bar represents 50 μm. **b** Representative immunofluorescence staining of selected protein targets in human testicular biopsies (from patients with complete spermatogenesis (iv–vi) and Sertoli cell only (vii–ix) compared to images from Human Protein Atlas (i–iii) and mouse testis (x–xii). All images at ×20 and scale bar represents 50 μm.

Next, immunolocalization was used to gauge the expression of the proteins FLNA, PSPC1, SREBP2 in the various testicular compartments or cell types (Fig. 6). FLNA was highly expressed in the cytoplasm of peritubular myoid cells, as well as expressed in elongating spermatids. PSPC-1 was expressed solely in germ cells notably in spermatocytes and round spermatids nuclei. The transcription factor, SREBP2 was expressed in the nuclei of somatic cells (Sertoli cell and Leydig cell nuclei) and in the cytoplasm of spermatocytes, round and elongating spermatids. Comparisons of immunostaining intensity between tissues from NC and HF mice revealed lower staining intensity of anti-FLNA in germ cells from the testis of HF mice compared to NC. In addition, testicular tissues from mice on HF had fewer PSPC-1 positive stained germ cells with smaller sized foci within the nuclei when compared to testis from mice on NC diet. Finally, a decreased staining intensity of SREBP2 was observed in HF diet tissues correlating with Western blot findings.

### Proteins altered by the HF diet are also expressed in the human testis

To ascertain whether the selected protein targets (FLNA, SREBP2, PSPC1) were expressed in the human testis and whether the same immunolocalisation pattern are observed, testicular samples were obtained from men undergoing testicular biopsy who either had complete spermatogenesis (CS) or had no germ cells present (Sertoli cell only, SCO). We confirmed the expression of FLNA, SREBP2 and PSPC-1 in human testicular tissues using the same antibodies which cross react with both species. The same staining patterns were seen as those in mouse testis suggesting expression within the same testicular cell types. In addition, staining patterns were fairly consistent using human testicular samples which were compared to the Human Protein Atlas repository (Fig. 6b).

In patients with CS, SREBP2 was expressed both in Sertoli and germ cells. In SCO samples, SREBP2 staining persisted in the nuclei of Sertoli cells. FLNA was observed in human germ cells of the CS samples but no staining was seen in SCO samples where no germ cells exist. Finally, PSPC-1 staining was also observed as paraspeckles within the germ cell nuclei of human CS samples and as expected for this germ cell-specific marker, no PSPC-1 expression was detected in SCO samples. Together, these experiments confirmed that in normal human testicular tissue (CS samples), FLNA, PSPC-1 and SREBP2 are expressed in the same cell types as observed in the mouse testis suggesting possible conserved roles for these proteins in both species.

## Discussion

There is an increasing prevalence of obesity in children and adults, and high consumption of dietary fats are associated with alterations in male fertility and hormonal dysregulation. High BMI is associated with low total sperm count [35] and around 65% of men with infertility have hyperlipidaemia [36]. It is also known that sperm volume and motility can be affected by elevated total cholesterol and low density lipoprotein [37]. Together, these observations suggest that abnormal lipid metabolism in the male reproductive system can affect fertility

The effects of high fat or obesity on semen parameters are increasingly described but the effects on the testis, an organ exquisitely sensitive to even subtle changes of whole body metabolism, has not been thoroughly studied [38] and key players and biological pathways remain elusive. Molecular perturbations within the testis in diet-induced obesity rodent models have been studied at the transcriptome level with modest changes in gene expression and effects on microRNAs and sperm epigenome [18, 22]. Another study of the rat testicular proteome in conjunction with long non-coding RNAs from HF diet has been published, with pathways relevant to cytoskeleton remodelling and anti-oxidative functions described [24]. A discovery study as described here, where careful histological assessment with the integration of proteomic data can provide greater insight into the consequences of HF diet.

Histological assessment confirmed marked distortion of the seminiferous tubules in the chronic HF diet-fed mice with a reduction in germ cell numbers, particularly spermatogonial stem cells and maturing spermatids with effects on meiotic index. The number of Sertoli cells, crucial for germ cell sustenance were reduced. Therefore, HF diet does not only impact germ cell development but also has an effect on Sertoli cells and the two are likely to be interrelated. A review of the proteomic data has provided insight into the biological pathways affected by HF diet in the testis. Since proteins do not function alone, a protein–protein interaction analysis showed high interaction scores suggesting engagement in a network of proteins with overlapping functions. IPA pathway analysis helped to identify the key roles of these proteins and further analysis of canonical pathways revealed the top deregulated pathways related to ‘cavelolar-mediated endocytosis signalling’, as well as effects on ‘Sertoli: germ cell signalling’. These pathways have particular relevance to the blood testis barrier (BTB), an androgen-regulated structure which is disrupted in animals exposed to a HF diet [17, 39].

The BTB divides the seminiferous tubules into a basal compartment (where early spermatogonia reside) and an adluminal compartment where post-meiotic cells are protected from toxic insults and secluded from the immune system [40]. The BTB forms a complex network of proteins acting as tight junctions (TJ) and gap junctions, together with desmosomes, integral membrane adaptor and cytoskeletal proteins. The BTB is dynamic as germ cells move between the two compartments with the assembly of a new ‘BTB’ [41] partly dependent on internalisation and recycling of integral membrane proteins using caveolar-mediated endocytosis. Since caveolae-associated protein 1 (PTRF/CAV1), filamins A/B and integrin alpha 11 were all significantly downregulated for mice on HF diet, we propose that these specific proteins are implicated in the disruption of the testicular junction. The consequences of this may affect the availability/recycling of proteins at the BTB and impact proper development of germ cells at later stages, in support of which a decline in germ cell numbers was also noted for mice on HF diet.

Although mass spectrometry is a powerful and insightful technique, the experiments conducted here were only focused on providing qualitative and quantitative information at the protein/peptide level. Additional information related to post-translational modifications and protein turnover for example, were not accounted for in the scope of this work. Complimentary Western blot studies were conducted on selected key proteins for further validation, based on the magnitude of fold change (compared to control mice), novelty and relevance to human testicular biology. All selected targets (AR, PSPC1, FLNA, APOAI, SREBP2) had conserved expression across species with high expression levels in both mouse and human testicular tissues, and similar immunolocalisation patterns.

Firstly, we were interested in the filamin family (subtypes filamin A, B and C) regulators of the developing BTB in the postnatal testis which form a crucial link between the cellular cytoskeleton and extracellular matrix and found in cell: cell junctions of endothelial cells and in those undergoing polarisation [42]. Filamins have a number of different biological roles with both filamin A and B downregulated in the testis of mice on HF diet. In the adult testis, filamin A and B are reported to be localised to the basal compartment [43] and important for caveolar-mediated endocytosis of TJ proteins at the BTB and in axonemal formation during ciliogenesis [40, 44–46]. Given that developing spermatids start to polarise and form an axoneme as part of the sperm tail, filamins may have a role here supported by the stage specific expression of filamin A in developing spermatids, and it was in this distribution where much of the filamin A signal was lost in the HF model.

Other proteins that interact with filamin A, include the AR and nephrocystin 4 (NPHP4) and we found in HF conditions, a significant decrease in filamin interacting protein NPHP4, important for cytoskeleton signalling, cell adhesion and ciliary development [47] and associated with human male infertility [48]. Filamin A also interacts with AR, facilitating its nuclear translocation and transactivation; it can also disrupt AR interdomain interactions [49, 50]. Downregulation of AR in mice fed a HF diet has been previously described and is required for normal fertility [17, 51]. In mice on HF diet, we find reduced testicular expression of AR and AR coactivator, PSPC-1 [52] which is expressed in nuclear paraspeckles as part of an interaction complex with other proteins acting as AR co-regulators in mouse Sertoli cells [52, 53]. Here in HF diet mice, reduced expression of PSPC-1 was observed in Sertoli cells, as well as stage specific expression of PSPC-1 in spermatocytes and round spermatids, which do not express AR. The role of PSPC-1 in these cell types has yet to be elucidated but may be engaged in gene regulation via RNA processing and DNA repair, and acting synergistically with many other transcription factors.

The final proteomic target selected for validation was SPATA-20 (also referred to as SSP411), a thioredoxin-like protein expressed in spermatids [54] and Ssp411−/− mice have reduced sperm numbers, motility and abnormal sperm morphology [55]. Thioredoxins catalyse protein disulphide bonds and regulate enzymes involved in antioxidant defence and transcription factors. SPATA-20 expression is increased 5-fold in spermatogenic cells from patients with type 1 diabetes, where a deregulation of oxidative genes is noted when compared to healthy controls [56]. SPATA-20 may have a protective role in sperm likely in the context of sperm oxidative stress which is well associated obesity [19]. In our study, increased SPATA-20 may represent a compensatory response to the potential oxidative stress of HF diet.

In summary, we used a high fat diet model that is representative of human fertility than genetic mouse models. This study focused mainly on the abnormalities that occurred at the protein level and as a result, we have identified several candidate proteins and conducted pathway analysis around the effects of HF diet on the testis providing novel insights not previously described. This is tissue level data and some changes in proteins may be due to a reduced number of a particular cell type and may warrant further interrogation to study specific cell types.

Here we show that many of the identified targets are also expressed in the human testis, it is possible that similar candidates are involved in human infertility and targets such as SREBP2 may be targeted therapeutically by readily available drugs such as metformin [57]. Our work provides a foundation for understanding how obesity may affect male fertility and future functional studies should be directed towards providing support for these potential players.

## Supplementary information

Supplemental information
