# Supplementary material for: High fat diet causes distinct aberrations in the testicular proteome
Source: Int J Obes (Lond). 2020 Jul 16;44(9):1958–69. doi: 10.1038/s41366-020-0595-6 (PMC7445115; doi:10.1038/s41366-020-0595-6)
Supplement: Supplementary file 1 — Supplemental information [file 41366_2020_595_MOESM1_ESM.docx]

**Supplementary Data: High fat diet causes distinct aberrations in the testicular proteome.**

**Supplementary methods**

***Histological analysis and immunostaining.***

All photomicrograph assessments were done using the software Image J® (Image Processing and Analysis in Java). After calibration, the seminiferous tubule diameter was determined with the ‘straight line selection’ tool using photomicrographs taken with 10x objective. Cells were counted from the photomicrographs obtained using a 40x objective and the ‘free hand selection’ tool used to delimit the area quantified. For these analyses, 25 fields/mouse were also assessed. 4 slides of sections cut from the middle of the testis were selected, and 3 areas from each slide were analysed for testicular cell composition. The number of Sertoli cells, types A and B spermatogonia, pachytene spermatocytes and round spermatids, steps 1 to 8 were counted to quantitate spermatogenesis ^26^. The nuclei of different germ cells were counted in 100 round seminiferous tubule cross sections, chosen at random for each mouse. Abercrombie correction for section thickness and differences in the nuclear or nucleolar diameter ^27^, and Amann modified correction ^28^ were applied to determine absolute cell number. Average number of cells = [number of nuclei counted x thickness (μm) of section]/[average diameter of nuclei (μm) + thickness (μm) of section]. Results of cell numbers per cross section of seminiferous tubule ^29^ were expressed as: coefficient of efficiency of spermatogonial mitosis = (pachytene spermatocytes)/(type A1 spermatogonia); meiotic index (rate of germ cell loss during meiosis) = (round spermatids)/(pachytene spermatocytes); and Sertoli efficiency = (round spermatids)/(Sertoli cell nuclei). Cellular counts of Sertoli cells, types A and B spermatogonia, and pachytene spermatocytes and round spermatids, steps 1 to 8 were undertaken as previously described ^26^.

**Immunofluorescence and confocal microscopy**

5μm sections were dewaxed and rehydrated in Histoclear (National Diagnostics, UK), 100% and 70% ethanol followed by antigen retrieval technique using microwaving for 10 min in 0.01 M citrate buffer, pH 6.0. Sections were preincubated with 1% blocking solution of goat serum for 1 hr at room temperature. Sections were then incubated with diluted primary antibodies all raised in rabbit at 1:200 dilution (anti-Filamin A, Paraspeckle protein-1 (Abcam, Cambridge, MA, USA) and SPATA-20 [1:200], (Atlas Antibodies 1:100) at RT for 1 hr in a humidified chamber. Sections were washed three times in PBS and incubated with secondary antibody (goat anti-rabbit Alexa Fluor**®** 488, Thermo Fisher) for 1 hr at room temperature. After three washes in PBS, sections were mounted with coverslips using ProLong Gold with DAPI (Thermo Fisher Scientific). Confocal microscopy was undertaken for visualization of immunostaining (Zeiss 510) and the same pinhole and detector gain settings were used when comparing staining intensity between testes from the two groups.

**Terminal deoxynucleotidyl transferase staining (TUNEL)**

Sections were dewaxed, boiled in 0.01M citrate buffer, pH 6.0 in the microwave and permeabilised with 0.1% triton X-100 (BDH). The TUNEL label was combined with 10% of TUNEL enzyme (terminal deoxynucleotidyl transferase; Roche) and 50µl applied to each section for 1 hour at 37ºC in a humidified chamber. Negative controls excluded the TUNEL enzyme.

***LC-MS configuration***

Samples were transferred with aqueous 0.1% (v/v) formic acid to the precolumn at a flow rate of 5 µl/min for 5 min. Mobile phase A consisted of water containing 0.1% (v/v) formic acid, whilst mobile phase B consisted of acetonitrile containing 0.1% (v/v) formic acid. After desalting and preconcentration, peptides were eluted from the pre-column to the analytical column with separation using a gradient of 3-40% mobile phase B for 90 min (flow rate of 300 nl/min), followed by a 2 min column rinse with 85% of mobile phase B. Re-equilibration of columns used initial conditions for a 20 minute period. Analytical column temperature was maintained at 35**°**C and lock mass compound, [Glu^1^]-Fibrinopeptide B (Sigma-Aldrich, St Louis, MO)(200 fmol/µl), was delivered by the auxiliary pump of the LC system at 500 nl/min to the reference sprayer of the NanoLockSpray source of the mass spectrometer.

MS analysis of tryptic peptides was performed using a Synapt G2-S*i* mass spectrometer (Waters Corporation, Wilmslow, UK). For all measurements, the mass spectrometer was operated in resolution-mode with nominal resolution of 25,000 FWHM. All analyses were performed in positive mode electrospray ionisation (ESI). The ion source block temperature and capillary voltage were set to 70ºC and 3.2 kV, respectively. The time of flight analyzer (ToF) of the mass spectrometer was externally calibrated with a NaCsI mixture from *m/z* 50 to 1990. The data were post-acquisition lock mass corrected using the doubly charged monoisotopic ion of [Glu^1^]-Fibrinopeptide B. The reference sprayer was sampled with a frequency of 60 s. Accurate mass LC-MS data were collected in mobility assisted data independent (LC-UDMS^E^) mode of acquisition ^31^.

**Supplementary Results**

**Supplementary figure** 1 (a) TUNEL analysis in a representative testicular section from mice on NC diet versus control. A testicular section from 4 month C57BL/6 mouse treated with Busulphan (known to lead to germ cell apoptosis) is shown and negative control (b) RT-qPCR analysis of apoptosis genes Caspase-3 and Parp-1 reveals no change in testicular gene expression levels between NC and HF fed mice (analysed using 2 tailed, unpaired t-test)

**a**

**
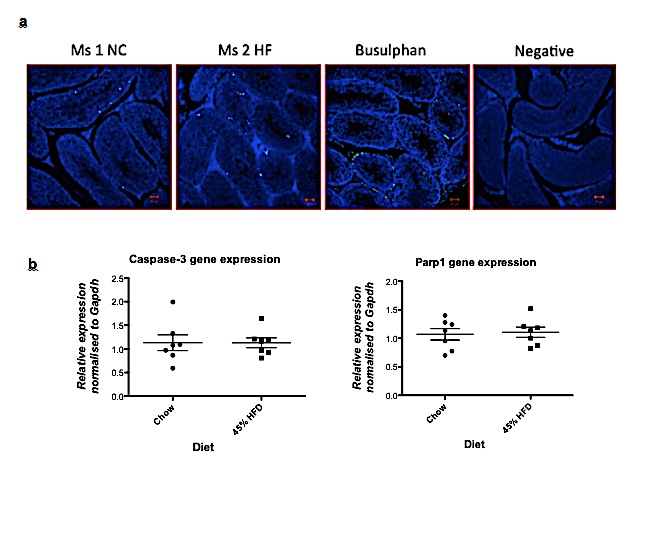
**

**b**

**Supplementary figure 2**. STRING database v 10.5 Protein: protein interactions between the top 102 differentially expressed proteins found in the HF conditions when compared to control. Edges represent protein: protein associations. The edge attributes include overall confidence score.


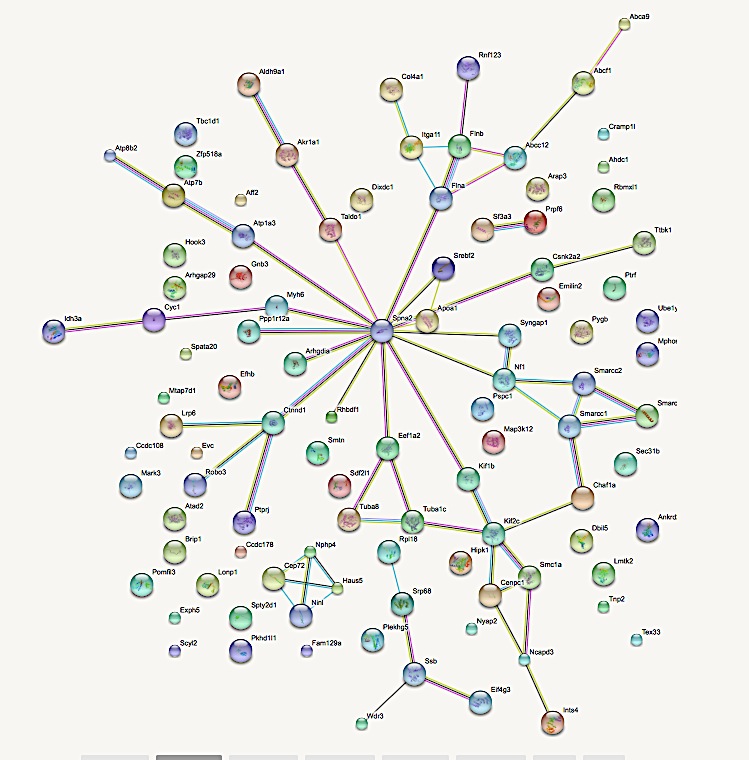


| **Network stats (STRING v10.5)** | |
| --- | --- |
| No of nodes | 100 |
| Number of edges | 65 |
| Expected number of edges | 45 |
| Average local clustering coefficient | 0.331 |
| PPI enrichment (p value) | ***0.0036 |

**Known Interactions**

| 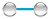 | from curated databases |
| --- | --- |
| 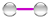 | experimentally determined |

**Predicted Interactions**

| 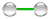 | gene neighborhood |
| --- | --- |
| 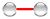 | gene fusions |
| 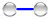 | gene co-occurrence |

**Others**

| 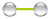 | textmining |
| --- | --- |
| 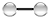 | co-expression |
| 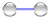 | protein homology |

Supplementary Table 1. **Histological differences between testicular tissues from mice on a HF versus NC diet using H&E sections.** A review of the histological features were assessed by the Histopathologist using 25 fields/animal (total 4 mice per group). *p<0.05, **p<0.01, ***p<0.001 by two tailed unpaired T-test

| **Table 1. Histological differences between testicular tissues from mice on a HF versus NC diet.** | | | |
| --- | --- | --- | --- |
| **Histological parameter** | **Normal Chow**  **(n=4 mice)**  **Mean [SD]** | **High Fat Diet**  **(n=4 mice)**  **Mean [SD]** | **P value** |
| **Tubule Diameter (um)** | **246.75 [59.54]** | **166.25 [29.35]** | ***0.05** |
| **Type A spermatogonia (n)** | **9.75 [1.71]** | **15.00 [2.58]** | ***0.01** |
| **Type B spermatogonia (n)** | **17.00 [5.60]** | **18.25 [5.19]** | **0.75** |
| **Mitotic index** | **6.81 [1.51]** | **5.20 [0.58]** | **0.05** |
| **Pachytene spermatocytes (n)** | **65.25 [12.37]** | **77.00 [4.69]** | **0.13** |
| **Round spermatids (n)** | **227.50 [16.20]** | **168.50 [20.98]** | ****0.004** |
| **Meiotic index** | **3.55 [0.49]** | **2.18 [0.15]** | *****0.0004** |
| **Sertoli cells (n)** | **15 [0.82]** | **9.75 [1.71]** | *****0.001** |

**Supplementary table 2.** Statistically significant differentially expressed proteins in testes from HF diet mice compared to NC (data presented as log2 FC)

| **Entry**  **Name** | **Protein Name** | **Log2**  **Fold change** |
| --- | --- | --- |
| \| CO4A1 \| \| --- \| \| LMTK2 \| \| PSPC1 \| \| EFHB \| \| GBB3 \| \| SCYL2 \| \| IDH3A \| \| SYGP1 \| \| SRP68 \| \| TTBK1 \| \| SRBP2 \| \| CRML \| \| FLNA \| \| RHG29 \| \| SMRC1 \| \| Z518A \| \| AT8B2 \| \| ABCA9 \| \| INT4 \| \| NPHP4 \| \| DBIL5 \| \| AFF2 \| \| SMAL1 \| \| NF1 \| \| M3K12 \| \| SF3A3 \| \| PTRF \| \| ATAD2 \| \| HAUS5 \| \| MA7D1 \| \| NIBAN \| \| CNDD3 \| \| LONM \| \| EXPH5 \| \| RMXL1 \| \| CC108 \| \| RHDF1 \| \| ALEX \| \| RL18 \| \| ROBO3 \| \| EMIL2 \| \| MARK3 \| \| CY1 \| \| RN123 \| \| GDIR1 \| \| SMRC2 \| \| SDF2L \| \| TBCD1 \| \| MYH6 \| \| IF4G3 \| \| CSK22 \| \| TALDO \| \| ITA11 \| \| PKHG5 \| \| HOOK3 \| \| NINL \| \| ABCF1 \| \| SMTN \| \| ANR24 \| \| AT1A3 \| \| APOA1 \| \| FLNB \| \| AHDC1 \| \| SMC1A \| \| NYAP2 \| \| AK1A1 \| \| PTPRJ \| \| EF1A2 \| \| SPT2 \| \| CEP72 \| \| KIF1B \| \| CC178 \| \| HIPK1 \| \| PKHL1 \| \| WDR3 \| \| PYGB \| \| ARAP3 \| \| TBA8 \| \| SC31B \| \| FANCJ \| \| CAF1A \| \| STP2 \| \| EVC \| \| AL9A1 \| \| KIF2C \| \| PRP6 \| \| UBA1Y \| \| TEX33 \| \| LRP6  MYPT1 \| \| NAV1 \| \| ATP7B \| \| DIXC1 \| \| CENPC \| \| SPT20 \| \| LA \| \| MRP9 \| \| SPTN1 \| \| CTND1 \| \| TBA1C \| \| YH010 \| \|  \| | \| Collagen alpha-1(IV) chain \| \| --- \| \| Serine/threonine-protein kinase LMTK2 \| \| Paraspeckle component 1 (Paraspeckle protein 1) \| \| EF-hand domain-containing family member B \| \| Guanine nucleotide-binding protein G(I)/G(S)/G(T) subunit beta-3 \| \| SCY1-like protein 2 (Coated vesicle-associated kinase of 104 kDa) \| \| Isocitrate dehydrogenase [NAD] subunit alpha, mitochondrial \| \| Ras/Rap GTPase-activating protein SynGAP (Neuronal RasGAP) \| \| Signal recognition particle subunit SRP68 (SRP68) \| \| Tau-tubulin kinase 1 \| \| Sterol regulatory element-binding protein 2 (SREBP-2) \| \| Protein cramped-like (Cramped chromatin regulator homolog 1) \| \| Filamin-A (FLN-A) (Actin-binding protein 280 \| \| Rho GTPase-activating protein 29 \| \| SWI/SNF complex subunit SMARCC1 complex 155 kDa subunit) \| \| Zinc finger protein 518A \| \| Phospholipid-transporting ATPase ID \| \| ATP-binding cassette sub-family A member 9 \| \| Integrator complex subunit 4 (Int4) \| \| Nephrocystin-4 (Nephroretinin) \| \| Diazepam-binding inhibitor-like 5 (Endozepine-like peptide) (ELP) \| \| AF4/FMR2 family member 2 (FMR2P) (Fragile X mental retardation protein  2 homolog) \| \| SWI/SNF-related matrix-associated actin-dependent regulator of chromatin  subfamily A-like protein 1 \| \| Neurofibromin (Neurofibromatosis-related protein NF-1) \| \| Mitogen-activated protein kinase kinase kinase 12 \| \| Splicing factor 3A subunit 3 (SF3a60) (Spliceosome-associated protein 61) \| \| Polymerase I and transcript release factor (Cav-p60) (Cavin-1) \| \| ATPase family AAA domain-containing protein 2 \| \| HAUS augmin-like complex subunit 5 \| \| MAP7 domain-containing protein 1 \| \| Protein Niban (Protein FAM129A) \| \| Condensin-2 complex subunit D3 (Non-SMC condensin II complex subunit D3) \| \| Lon protease homolog, mitochondrial \| \| Exophilin-5 \| \| RNA binding motif protein \| \| Coiled-coil domain-containing protein 108 \| \| Inactive rhomboid protein 1 \| \| Protein ALEX (Alternative gene product encoded by XL-exon) \| \| 60S ribosomal protein L18 \| \| Roundabout homolog 3 \| \| EMILIN-2 (Basilin) \| \| MAP/microtubule affinity-regulating kinase 3 \| \| Cytochrome c1, heme protein, mitochondrial (Complex III subunit 4) \| \| E3 ubiquitin-protein ligase RNF123 \| \| Rho GDP-dissociation inhibitor 1 (Rho GDI1a) \| \| SWI/SNF complex subunit SMARCC2 (BRG1-associated factor 170) \| \| Stromal cell-derived factor 2-like protein 1 (SDF2-like protein 1) \| \| TBC1 domain family member 1 \| \| Myosin-6 (Myosin heavy chain 6) \| \| Eukaryotic translation initiation factor 4 gamma 3 \| \| Casein kinase II subunit alpha \| \| Transaldolase \| \| Integrin alpha-11 \| \| Pleckstrin homology domain-containing family G member 5 \| \| Protein Hook homolog 3 (mHK3) \| \| Ninein-like protein \| \| ATP-binding cassette sub-family F member 1 \| \| Smoothelin \| \| Ankyrin repeat domain-containing protein 24 \| \| Sodium/potassium-transporting ATPase subunit alpha-3 \| \| Apolipoprotein A-I (Apo-AI) \| \| Filamin-B (FLN-B) (ABP-280-like protein) \| \| AT-hook DNA-binding motif-containing protein 1 \| \| Structural maintenance of chromosomes protein 1A (SMC protein 1A) \| \| Neuronal tyrosine-phosphorylated phosphoinositide-3-kinase adapter 2 \| \| Aldo-keto reductase family 1 member A1 \| \| Receptor-type tyrosine-protein phosphatase \| \| Elongation factor 1-alpha 2 (EF-1-alpha-2) \| \| Protein SPT2 homolog (SPT2 domain-containing protein 1) \| \| Centrosomal protein of 72 kDa (Cep72) \| \| Kinesin-like protein KIF1B \| \| Coiled-coil domain-containing protein 178 \| \| Homeodomain-interacting protein kinase 1 \| \| Fibrocystin-L (Polycystic kidney and hepatic disease 1-like protein 1) \| \| WD repeat-containing protein 3 \| \| Glycogen phosphorylase, brain form \| \| Arf-GAP with Rho-GAP domain, ANK repeat and PH domain-containing protein \| \| Tubulin alpha-8 chain \| \| Protein transport protein Sec31B (SEC31-like protein 2) \| \| Fanconi anemia group J protein homolog (Protein FACJ) \| \| Chromatin assembly factor 1 subunit A (CAF-1 subunit A) \| \| Nuclear transition protein 2 (TP-2) (TP2) \| \| Ellis-van Creveld syndrome protein homolog \| \| 4-trimethylaminobutyraldehyde dehydrogenase \| \| Kinesin-like protein KIF2C \| \| Pre mRNA processing factor 6  Ubiquitin like modifier activating enzyme 1 \| \| Testis expressed sequence 33 \| \| Low-density lipoprotein receptor-related protein 6 (LRP-6) \| \| Protein phosphatase 1 regulatory subunit 12A \| \| Neuron navigator 1 \| \| Copper-transporting ATPase 2 \| \| Dixin1 \| \| Centromere protein C \| \| Spermatogenesis-associated protein 20 \| \| Lupus La protein homolog \| \| Multidrug resistance-associated protein 9 \| \| Spectrin alpha chain, non-erythrocytic 1 \| \| Catenin delta-1 \| \| Tubulin alpha-1C chain \| \| Putative IQ motif and ankyrin repeat domain-containing protein \| \|  \| | \| -2.34 \| \| --- \| \| -1.85 \| \| -1.77 \| \| -1.71 \| \| -1.60 \| \| -1.59 \| \| -1.53 \| \| -1.53 \| \| -1.52 \| \| -1.52 \| \| -1.50 \| \| -1.49 \| \| -1.48 \| \| -1.48 \| \| -1.43 \| \| -1.42 \| \| -1.40 \| \| -1.38 \| \| -1.36 \| \| -1.34 \| \| -1.33 \| \| -1.31 \| \| -1.31 \| \| -1.30 \| \| -1.30 \| \| -1.29 \| \| -1.29 \| \| -1.29 \| \| -1.28 \| \| -1.27 \| \| -1.27 \| \| -1.26 \| \| -1.25 \| \| -1.25 \| \| -1.25 \| \| -1.25 \| \| -1.25 \| \| -1.24 \| \| -1.21 \| \| -1.21 \| \| -1.21  -1.21 \| \| -1.20 \| \| -1.19 \| \| -1.18 \| \| -1.18 \| \| -1.17 \| \| -1.16 \| \| -1.15 \| \| -1.15 \| \| -1.14 \| \| -1.14 \| \| -1.14 \| \| -1.14 \| \| -1.12 \| \| -1.11 \| \| -1.11 \| \| -1.11 \| \| -1.10 \| \| -1.10 \| \| -1.09 \| \| -1.09 \| \| -1.08 \| \| -1.07 \| \| -1.07 \| \| -1.07 \| \| -1.07 \| \| -1.07 \| \| -1.07 \| \| -1.06 \| \| -1.05 \| \| -1.05 \| \| -1.05 \| \| -1.04 \| \| -1.04 \| \| -1.04 \| \| -1.04 \| \| -1.04 \| \| -1.04 \| \| -1.04 \| \| -1.04 \| \| -1.02 \| \| -1.01 \| \| -1.00 \| \| -1.00 \| \| +1.02 \| \| +1.03 \| \| +1.03 \| \| +1.08 \| \| +1.08  +1.08 \| \| +1.10 \| \| +1.19 \| \| +1.16 \| \| +1.18 \| \| +1.20 \| \| +1.21 \| \| +1.22 \| \| +1.22 \| \| +1.25 \| \| +1.27 \| \| +1.32 \| \| +1.39 \| |

Supplementary table 3. **. Functional categories of identified proteins differentially expressed in HF diet**

| **Table 2. GO terms for differentially expressed proteins** | | | |
| --- | --- | --- | --- |
| **PANTHER GO Slim Biological process** | **#** | **+/-** | **P value** |
| **Multi-cellular organism process** | **6** | **+** | **4.45E-03** |
| **Chromosome segregation** | **5** | **+** | **7.32 E-05** |
| **Meiosis** | **2** | **+** | **2.05 E-02** |
| **RNA splicing** | **3** | **+** | **2.74E-02** |
| **Mitosis** | **6** | **+** | **2.82 E-03** |
| **Cellular component movement** | **5** | **+** | **1.51 E-02** |
| **Catabolic process** | **13** | **+** | **1.60E-04** |
| **Cellular component morphogenesis** | **8** | **+** | **4.14 E-02** |
| **Cellular component biogenesis** | **17** | **+** | **1.48E-03** |
| **Protein transport** | **8** | **+** | **4.14 E-02** |
| **Nitrogen compound metabolic process** | **17** | **+** | **9.89 E-03** |

Supplementary table 4. Differentially expressed proteins found in both NC and HF from Mass Spectrometry selected for Western blot validation.
